# Nomogram Including Neutrophil-to-Lymphocyte Ratio for the Prediction of Stroke-Associated Infections

**DOI:** 10.3389/fneur.2020.574280

**Published:** 2020-11-02

**Authors:** Yan Lan, Wenzhe Sun, Yuxi Chen, Jinfeng Miao, Guo Li, Xiuli Qiu, Xiaoyan Song, Xin Zhao, Zhou Zhu, Yebin Fan, Suiqiang Zhu

**Affiliations:** ^1^Department of Neurology, Tongji Hospital, Tongji Medical College, Huazhong University of Science and Technology, Wuhan, China; ^2^Solomon H. Snyder Department of Neuroscience, Johns Hopkins University, Baltimore, MD, United States; ^3^School of Computer Science and Technology, Huazhong University of Science and Technology, Wuhan, China

**Keywords:** stroke, nomogram, infection, neutrophil-to-lymphocyte ratio (NLR), China

## Abstract

Stroke has been a leading cause of mortality in China. Stroke-associated infections (SAI) are common complications, occurring in 5–65% of stroke patients. Faced with SAI, clinicians often are placed in a considerable dilemma. On the one hand, preventive overuse of antibiotics will lead to the emergence of drug-resistant bacteria. On the other hand, treatment delay of the infection will likely result in a poor outcome. Therefore, it is necessary to determine the early predictors of post-stroke infection to screen patients with high infection risk for early clinical intervention, thereby promoting and improving survival rates. We assessed 257 patients with acute ischemic stroke from a consecutive retrospective cohort. Data of these patients were obtained from three hospitals (TongJi Hospital and its two branches) between August 2018 and June 2019. Of these patients, 59 (23.0%) developed SAI. SAI was defined according to the modified Centers for Disease Control and Prevention criteria. There were 38 patients (64.4%) who developed pneumonia, 11 with urinary tract infections (18.6%), and 10 with other infections (16.9%). We found that a higher neutrophil-to-lymphocyte ratio (adjusted odds ratio [aOR] = 1.16; 95% confidence interval [CI], 1.01–1.33; *P* = 0.034), National Institutes of Health Stroke Scale score (aOR = 1.18; CI, 1.09–1.27; *p* < 0.001), and dysphagia (aOR = 2.95; CI, 1.40–6.22; *P* = 0.004) were risk factors for SAI. Of note, hypertriglyceridemia (aOR = 0.35; CI, 0.13–0.90; *P* = 0.029) was a protective factor, lowering the risk of SAI. To this end, a reliable nomogram was constructed for the prediction of SAI in our study (mean C-index value ± standard deviation = 0.821 ± 0.03). It has the potential to be widely used and may help identify patients at high risk for SAI and make timely clinical decisions. Given our study was based on relatively small dataset, the results should be interpreted with care and external validation in independent datasets is very necessary.

## Introduction

Stroke is a debilitating disease and has been a leading cause of mortality in China. It is imperative to conduct clinical studies in search of strategies to reduce complications and increase the survival rate among stroke patients. Stroke-associated infections (SAI), such as pneumonia and urinary tract infection (UTI), are common complications, occurring in 5–65% of stroke patients (1). Numerous evidence has demonstrated that SAI increases the occurrence of severe disabilities and mortality and lengthens hospitalization duration (2–4). Faced with SAI, clinicians often are placed in a considerable dilemma. On the one hand, preventive overuse of antibiotics will lead to the emergence of drug-resistant bacteria. On the other hand, treatment delay of the infection will likely result in a poor outcome. Therefore, it is necessary to determine the early predictors of post-stroke infection to screen patients with high infection risk for early clinical intervention, thereby promoting and improving survival rates.

Early studies identified several risk factors for SAI, such as an older age, the male sex, diabetes mellitus, hypertension, chronic obstructive pulmonary disease, stroke severity, and dysphagia (5, 6). Besides, recent studies have demonstrated that stroke-induced immunosuppression is another major cause of infections in patients with stroke (7, 8), causing the downregulation of systemic immune responses with a decrease in peripheral blood lymphocyte count or an increase in neutrophil-to-lymphocyte ratio (NLR) (7, 9). However, vital parameters, such as the length of the follow-up period, baseline risk factors, and clinical definitions, vary significantly (1). There is not enough research based in China, a country often considered to have a high incidence of antibiotic abuse and nosocomial infections (10, 11). Hence, determining objective and easily obtainable indicators to predict SAI in stroke patients in China is of essence.

In this multi-center retrospective study, our goal was to investigate the predictors associated with SAI in patients after stroke and to establish a convenient and accurate predictive nomogram of SAI risk in patients, calculating a probabilistic estimate to guide clinical decisions.

## Materials and Methods

### Patient Selection

Our study was conducted at three independent hospitals (TongJi Hospital and its two branches) between August 2018 and June 2019. Patients with acute ischemic stroke who were admitted within 7 days of symptom onset were recruited into the study (*n* = 359). All participants involved in this study provided written informed consent according to the Declaration of Helsinki, which was approved by the Ethics Committee of Tongji Medical College, Huazhong University of Science and Technology (ID:TJ-IRB20171108). The diagnosis of a stroke was confirmed by a neurologist based on computed tomography or magnetic resonance imaging findings and relevant symptoms. Imaging examinations and venous blood tests in the fasted state were conducted within 24 h after admission.

**Figure 1:**
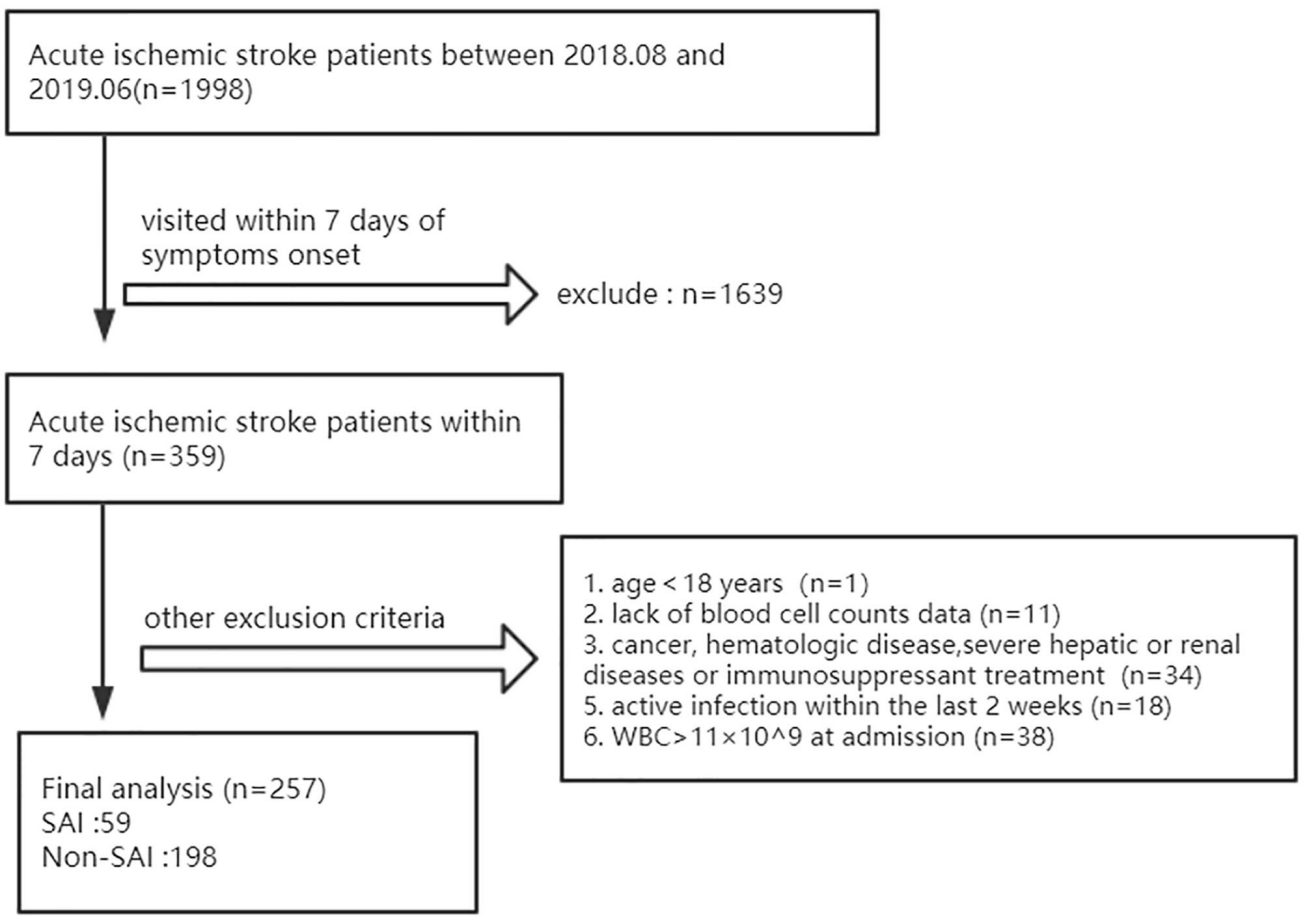
Enrollment flow chart of study cohort.

The exclusion criteria were as follows: age <18 years, lack of blood cell data, a history of cancer, hematologic disease or ongoing immunosuppressant treatment, severe hepatic or renal diseases, white blood cell count (WBC) > 11 × 10^9^ /L at admission, or active infection within the last 2 weeks. After evaluation, we enrolled 257 patients for analysis, including 59 with SAI (Figure 1).

In this study, SAI was diagnosed by the treating physician according to the Centers for Disease Control and Prevention criteria during the first 7 days after stoke onset (12, 13). Patients with infection after stroke must meet these exact standards, and the diagnoses were divided into the following three types: pneumonia, UTI, and other infections. Pneumonia was defined as the presence of relevant respiratory clinical symptoms and/or signs, with at least one of the following: leukocytosis (>11 × 10^9^ cells/L), fever (temperature ≥ 38.0°C), or a positive chest radiograph. UTI was defined based on the presence of relevant clinical symptoms and/or signs with positive microbiological cultures, or negative cultures with leukocytosis, and/or fever. The “other infections” group included patients with fever combined with leukocytosis who did not meet the pneumonia or UTI diagnosis criteria (14–16).

### Parameter Acquisition

The following sociodemographic, clinical, and laboratory data were used in our analysis: age, sex, comorbidities (hypertension, diabetes, hyperlipidemia, atrial fibrillation), current smoker and/or drinker status (within the last 6 months), use of thrombolysis, stroke severity upon admission based on the National Institutes of Health Stroke Scale (NIHSS) score and the modified Rankin Scale (mRS) score, presence of dysphagia (based on the water swallow test), and blood indices such as total homocysteine (tHCY), blood cell counts, lipid level, blood glucose, NLR, and lymphocyte subsets counts. The NLR was calculated by dividing the absolute neutrophil count by the absolute lymphocyte count. Hyperlipidemia was defined as hypercholesterolemia (fasting plasma cholesterol level ≥ 200 mg/dL) and hypertriglyceridemia (fasting plasma triglyceride level ≥ 150 mg/dL) based on the blood-lipid level (17, 18). All blood tests were conducted within 24 h of admission.

### Statistical Analyses

Continuous data are presented as medians and interquartile ranges and compared using the Mann–Whitney *U*-test. Categorical data are presented as frequencies and proportions, and the chi-square test and Fisher's exact test were used to compare these variables. Variables that were easily obtained in clinical practice were included for the derivation of the model predicting the SAI risk based on previous literature. Variables with *P* < 0.10 in univariate analysis results were subjected to multivariable logistic regression analysis in a stepwise manner. Statistical analyses to identify predictors were performed using Statistical Package for the Social Sciences version 22.0 (SPSS, Chicago, IL). In addition, the nomogram was formulated based on these predictors in multivariate analysis using the package “rms” in R (version 3.5.2, http://www.r-project.org/). Validation of the nomogram was performed based on its discrimination and calibration abilities. The concordance index [equivalent to the area under the curve [AUC] value, presented as C-index ± standard deviation] reflected the model's discrimination ability, wherein higher the C-index, the better the nomogram's accuracy to identify in-hospital patients' SAI risk. Calibration curves were constructed to evaluate the model's predictive accuracy by comparing the predicted probability with the observed probability in our study. The calibration curve was deemed appropriate if dots on the calibration plot were close to a 45° diagonal line. These calibration activities were conducted using bootstraps with 1,000 resamples.

## Results

The study included 257 patients with acute ischemic stroke, with 59 (23.0%) patients developing SAI. Of those with SAI, there were 38 patients (64.4%) who developed pneumonia, 11 with UTIs (18.6%), and 10 with other infections (16.9%). Table 1 presents the baseline characteristics of SAI patients and non-SAI patients. The frequency of dysphagia (*P* < 0.001) and atrial fibrillation (*P* = 0.002) was higher in SAI patients. Meanwhile, patients with SAI tended to have higher NIHSS (*P* < 0.001) and mRS (*P* < 0.001) scores, leukocyte counts (*P* = 0.041), neutrophil counts (*P* = 0.001), and NLR (*P* < 0.001) on admission. However, lymphocyte (*P* = 0.001), CD4+ T cell counts (*P* < 0.001), CD8+ T cell counts (*P* = 0.011) and hypertriglyceridemia levels (*P* = 0.004) were lower and more infrequent in those with SAI.

**Table 1:** Comparison of baseline characteristics between SAI and Non-SAI.

	**Non-saI (*n* = 198)**	**SaI (*n* = 59)**	**Univariate analysis**	**Multivariate analysis**		
			***P*-value**	***P*-value**	**OR**	**95%CI**
Sex, female (*n*, %)	51 (25.76%)	17 (28.81%)	0.640			
Age, years, median (IQR)	60 (50.25–66)	61 (52.5–70.5)	0.132			
Diabetes (*n*, %)	58 (29.29%)	16 (27.12%)	0.746			
Hypertension (*n*, %)	144 (72.73%)	42 (71.19%)	0.816			
Smoking (*n*, %)	87 (43.94%)	23 (38.98%)	0.499			
Drinking (*n*, %)	73 (36.87%)	19 (32.20%)	0.512			
Dysphagia (*n*,%)	27 (13.64%)	26 (44.07%)	<0.001*	0.004*	2.95	1.40–6.22
NIHSS, median (IQR)	2 (1–5)	7 (4–12)	<0.001*	<0.001*	1.18	1.09–1.27
MRS, median (IQR)	1 (1–2)	3 (2–4)	<0.001*			
Hypercholesterolemia (*n*, %)	17 (8.59%)	3 (5.08%)	0.580			
Hypertriglyceridemia (*n*, %)	57 (28.79%)	6 (10.17%)	0.004*	0.029*	0.35	0.13–0.90
Thrombolysis (*n*, %)			0.075			
Intravenous	6 (3%)	6 (10.17%)				
Intra–arterial	1 (0.51%)	1 (1.69%)				
HCY, μmol/L, median (IQR)	13.55 (10.9–17)	13.9 (10.35–17.5)	0.921			
Leukocyte, × 10^3^/μL, median (IQR)	6.49 (5.43–7.85)	7.08 (5.39–9.52)	0.041*			
Neutrophil, × 10^3^/μL, median (IQR)	4.02 (3.16–5.08)	4.99 (3.60–6.92)	0.001*			
Lymphocyte, × 10^3^/μL, median (IQR)	1.66 (1.31–2.08)	1.35 (1.07–1.79)	0.001*			
NLR, median (IQR)	2.38 (1.74–3.35)	3.9 (2.57–5.52)	<0.001*	0.034*	1.16	1.01–1.33
CD4+T, /μL, median (IQR)	653.66 (491.25–845.75)	500 (393–592)	<0.001*			
CD8+T, /μL, median (IQR)	304.50 (215.75–404)	251 (181–329.50)	0.011*			
NK, /μL, median (IQR)	189.50 (123–279.50)	168 (96–244)	0.052			
Th/Ts, median (IQR)	2.27 (1.73–2.85)	1.95 (1.60–2.65)	0.062			
Atrial fibrillation (*n*,%)	5 (2.52%)	8 (13.56%)	0.002*			
Admission blood glucose, median (IQR), mmol/L	5.43 (4.95–6.29)	5.69 (5.21–6.55)	0.066			

OR, Odds Ratio; CI, Confidence Interval; Variables with P < 0.10 in univariate analysis were included in multivariable logistic regression models for adjustment; *P < 0.05.

*Multivariable logistic regression adjusted for age, sex, stroke risk factors, thrombolysis, Hypercholesteremia, Hypertriglyceridemia, CD4+ T cell counts, NK cell counts, Th/Ts, NLR, admission NIHSS score, mRS score, and blood glucose, and Dysphagia*.

In the multivariable logistic regression analysis, NLR (adjusted odds ratio [aOR] = 1.16; 95% confidence interval [CI], 1.01–1.33; *P* = 0.034) and hypertriglyceridemia (aOR = 0.35; CI, 0.13–0.90; *P* = 0.029) remained significant after adjusting for confounders (Table 1). Besides, dysphagia (aOR = 2.95; CI, 1.40–6.22; *P* = 0.004) and NIHSS scores (aOR = 1.18; CI, 1.09–1.27; *p* < 0.001) were also significant. These variables were independent of each other.

**Figure 2:**
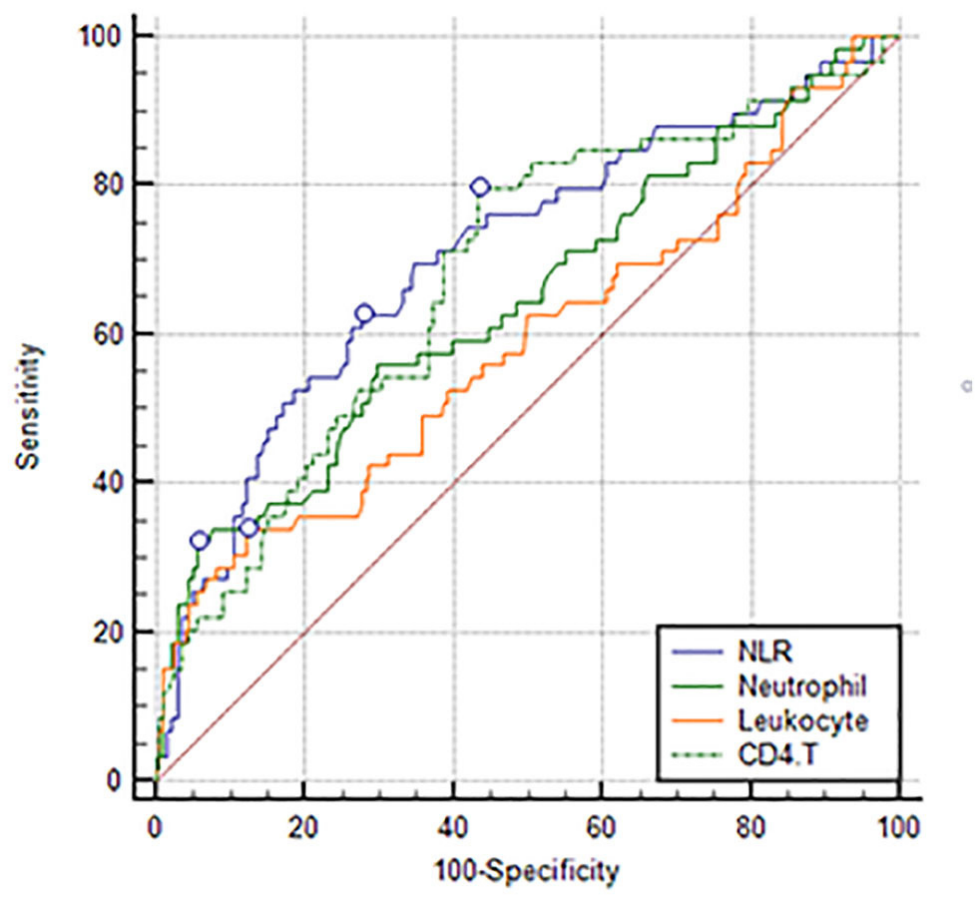
Predictive power comparison between NLR and other inflammatory markers.

Comparing the predictive power among NLR, the newly discovered index: CD4+ T cell count, and conventional inflammatory markers, the study demonstrated that NLR (0.707 [0.647–0.762]) had a higher AUC than leukocyte (0.588 [0.525–0.648]), neutrophil (0.646 [0.584–0.704]), and CD4+ T cell (0.680 [0.619–0.736]) counts (Figure 2). Although we did not find a significant difference between NLR and CD4+ T cell counts, the difference between NLR and leukocyte and neutrophil counts was significant.

**Figure 3:**
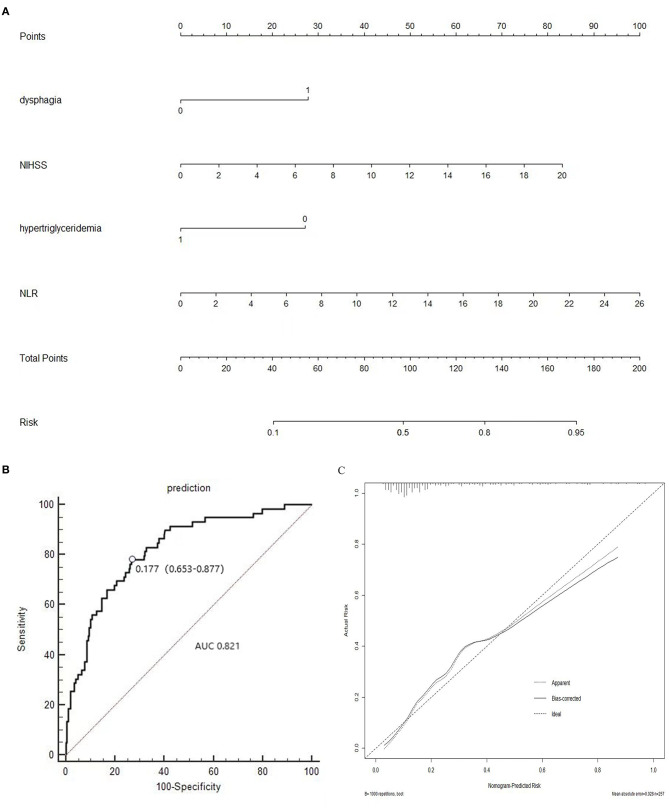
**(A)** Nomogram of the study population to predict SAI in patients after stroke; **(B)** Area under the receiver operating characteristic curve (AUROC), which is representative of predictive accuracy. The c-index value for the nomogram is 0.821 ± 0.03. **(C)** Calibration curve for the nomogram.

In addition, all the independent predictors identified in the multivariate logistic regression analysis of SAI were considered while constructing the nomogram (Figure 3A). Each predictor was projected upward to obtain the matching points on the small ruler (points) and the points assigned to the corresponding predictors were added up to obtain the total points. Then, a vertical line was drawn down from the “Total points” axis to the “risk” axis, and the corresponding risk of SAI was obtained. We then assessed the discrimination accuracy of this forecasting model. The Harrell's mean c-index value (± standard deviation) for the nomogram was 0.821 ± 0.03 (Figure 3B). With a cut-off value of 0.1768, the sensitivity and the specificity were 78.0 and 72.3%, respectively. Furthermore, the calibration curves for this nomogram demonstrated an excellent agreement between the predicted probability of SAI and the actual observations, with data points on the plots near to the 45° diagonal line (Figure 3C). This nomogram can predict the risk of infection after stroke individually according to the distinct conditions of different patients.

## Discussion

In this retrospective multi-center study, we analyzed the data of 257 acute ischemic stroke patients for SAI, which occurred in 23% of the patients. We found that a higher NLR, higher NIHSS score, and dysphagia were risk factors for SAI. Of note, hypertriglyceridemia was a protective factor lowering the risk of SAI.

The NIHSS score is often used as the measure of stroke severity upon admission. Consistent with the previous studies (5, 6, 19, 20), we demonstrated that the NIHSS score serves as a strong risk factor for infection after acute ischemic stroke. Patients with a high NIHSS score tended to have a high level of unconsciousness, thus spent most of their time resting in bed, and tended to be susceptible to aspiration pneumonia caused by gastroesophageal reflux. In addition, dysphagia promotes aspiration, causing an increased risk of pneumonia (21), thereby making it a prominent cause of SAI. Although tube feeding is known to be effective and relatively safe in supplying nutrition and reducing aspiration pneumonia in patients with dysphagia in the early stages of stroke, some research has demonstrated that it may be related to respiratory complications (22, 23). Therefore, early screening and treatment of dysphagia are recommended as effective measures to reduce the frequency of pneumonia after stroke (24).

In recent years, several studies have found that a high NLR is associated with SAI (9, 25). In a two-center retrospective cohort study (*n* = 1,317), a high NLR was observed to be associated with pneumonia after acute ischemic stroke. Furthermore, it was shown that NLR is also associated with pneumonia severity and clinical outcomes (9). Another recent study verified that a higher NLR is also associated with stroke severity on admission (26). In addition, a high NLR on admission correlated with a poor outcome 3 months after acute ischemic stroke (27). Thus, the association between NLR and SAI might be based on the severity of the stroke. An elevated NLR highly correlates with a severe and large volume of stroke cases (25–27), which usually emerge with a devastating NIHSS or mRS score. It is well-known that NIHSS and mRS scores are strong predictors of SAI (5, 9, 21). Another explanation for the correlation between NLR and SAI may be the changes in the immune system after acute ischemic stroke. Immunosuppression and systemic inflammation are known to be major contributors in the pathophysiology of ischemic stroke (28). In the inflammatory immune response after anoxia and ischemic injury of stroke, neutrophils are increased by demargination, delayed apoptosis, and stimulation of growth factors. At the same time, margination, redistribution, and accelerated apoptosis result in the decrease of protective lymphocytes like regulatory T cells and B cells (29–31). Besides, the shift from the pro inflammatory Th-1 type response to the anti-inflammatory Th-2 type response in T cells may increase the susceptibility to infections (32, 33). Our study provides further evidence that a higher NLR is an excellent predictor of SAI.

Thus, NLR is a good parameter reflecting not only stroke severity but also the body's immunity. Notably, it is the combination of well-recognized traditional inflammatory markers: neutrophilia and lymphocytopenia (8, 29, 34). Hence, NLR was found to be a better predictor than leukocyte and neutrophil counts in their predictive ability, compared with conventional inflammation markers and NLR in our study, with a significantly higher AUC of the NLR ROC curve. Although NLR shows no significant advantages when compared with CD4+ T cells, it appears to be an easier and more feasible clinical indicator.

Hypertriglyceridemia has been known to contribute to endothelial dysfunction and atherosclerosis, which could in turn contribute to ischemic stroke (35, 36). Furthermore, extremely high triglyceride levels may lead to pancreatitis (37). A meta-analysis of 10 trials and 77 917 high ischemic event risk individuals demonstrated that reducing blood triglyceride levels with omega-3 fatty acid had no significant clinical benefit in preventing major vascular events (38). Nevertheless, triglyceride was a protective factor for SAI in our study. This result was consistent with that in a previous retrospective cohort study (*n* = 1,491), where hyperlipidemia was associated with a decreased incidence of pulmonary infection (39). The participation of adipokines may be one possible explanation. Elevated plasma triglyceride levels are often associated with adipose tissue dysfunction. Adipocyte hypertrophy leads to adipocyte functional changes and a disorder of adipokines, including interleukin (IL)-6, tumor necrosis factor (TNF)-α, leptin, and adiponectin with proinflammatory and anti-inflammatory activities (40–42). In addition, obesity results in an increased influx of macrophages in the adipose tissue, and the M2 macrophages in humans have been demonstrated to have both pro inflammatory and anti-inflammatory properties (42, 43). In contrast, in a review of adipokines in cardiovascular diseases, anti-inflammatory adipokine adiponectin was thought to be decreased by obesity (44) and promote systemic metabolic dysfunction (45). Another explanation is avoidance of malnutrition, which is usually accompanied by dysbiosis and disturbances in normal barrier functions, leading to susceptibility to invasive infections (46, 47). Although the protective mechanism of hypertriglyceridemia against infections needs to be further elucidated, we have explored a new modality in the clinical management of patients with hypertriglyceridemia after stroke.

SAIs have attracted more attention in recent years, owing to its increased medical costs and unfavorable clinical outcomes. There are several predictive factors and scoring systems established in previous studies (5, 6, 48). For instance, the A2DS2-score and AIS-APS system are simple and valid clinical tools to predict infection risk after acute ischemic stroke from a large sample. However, the A2DS2 score, which was derived from multiple centers with a sample size in thousands, did not assess biological indicators. The AIS-APS score was derived from a prospective cohort and assessed the age, medical history, pre-stroke dependence, NIHSS score upon admission, Glasgow Coma Scale (GCS) score, symptom of dysphagia, Oxfordshire Community Stroke Project subtype, and blood glucose levels. The benefits of AIS-APS were its high simplicity and accuracy in clinical practice and its external validation. While most included variables in AIS-APS are clinical symptoms and demographic characteristics, we have emphasized on serum biological indicators and variables associated with systematic immunity in our model.

Herein, a reliable and convenient nomogram was constructed for the prediction of SAI. Nomograms are widely used in medicine and oncology for predicting the possibility of clinical events by integrating different variables, and they have merits like straightforward digital interface, adequate accuracy, and readily comprehensible prognoses (49). In recent years, it has become increasingly popular to apply a nomogram for stroke prognosis to help clinicians make sound and timely decisions. The nomogram in our study combined serum biological indicators and clinical symptoms for clinical prediction. Each predictor value has associated points on a scale, with the total points corresponding to a predicted SAI risk. Furthermore, to the best of our knowledge, our study is the first to construct a nomogram that included NLR for the prediction of SAI.

There are several limitations to this study that need to be addressed. First, although we recruited patients from three centers, the sample size was still small. While excluding patients with a WBC count > 11 on admission reduced the possibility of including patients with infections before admission, it may have led to the exclusion of some severe stroke patients because an elevated leukocyte count in the acute phase of ischemia stroke may be induced by severe stroke (50, 51). Thus, patients included in our study often with relatively mild strokes, the possibility of selection bias is inevitable. Second, there was no thrombectomy performed among the 257 patients in our study, likely because patients with a high WBC count were excluded in this study. This may have led to the exclusion of patients with thrombectomy and high NIHSS scores. Another possible limitation is that the Tongji hospital is a large regional referral hospital, and the time window used may have been missed when patients were transferred to our hospital. While patients in our study may lack adequate representativeness, our research provides ideas to other regional referral hospitals. Third, although only 2% of data (e.g., blood data) was missing in our study, information bias may also have occurred when the average interpolation was used. Finally, our study was a retrospective study and we were unable to verify our results through an external validation, therefore the reported nomogram needs validation in independent datasets and further prospective studies and fundamental research are needed, too.

In summary, our data demonstrated that dysphagia, the NIHSS score, NLR, and hypertriglyceridemia are associated with SAI. The nomogram constructed from these data may be a promising tool for patients after stroke to predict SAI. However, further investigations, from basic science to clinical investigation, are required to confirm these findings.

## Data Availability Statement

The datasets presented in this article are not readily available because further data mining is ongoing. Requests to access the datasets should be directed to lanyanswag@163.com.

## Ethics Statement

The studies involving human participants were reviewed and approved by the Ethics Committee of Tongji Medical College Huazhong University of Science and Technology. The patients/participants provided their written informed consent to participate in this study.

## Author Contributions

YL, WS, JM, GL, and XS: collected, analyzed and interpreted the data, and drafted the manuscript. XQ and XZ: analyzed imaging data. YC and ZZ: designed the study and revised the manuscript. All authors contributed to the article and approved the submitted version.

## Conflict of Interest

The authors declare that the research was conducted in the absence of any commercial or financial relationships that could be construed as a potential conflict of interest.

## References

[1] Vermeij FH Reimer WJMSO de Man P van Oostenbrugge RJ Franke CL de Jong G. Stroke-associated infection is an independent risk factor for poor outcome after acute ischemic stroke: data from the netherlands stroke survey. Cerebrovasc Dis. (2009) 27:465–71. 10.1159/000210093 19329851

[2] Katzan IL Cebul RD Husak SH Dawson NV Baker DW. The effect of pneumonia on mortality among patients hospitalized for acute stroke. Neurology. (2003) 60:620–5. 10.1212/01.WNL.0000046586.38284.60 12601102

[3] Kwan J Hand P. Infection after acute stroke is associated with poor short-term outcome. Acta Neurol Scand. (2007) 115:331–8. 10.1111/j.1600-0404.2006.00783.x 17489944

[4] Spratt N Wang Y Levi C Ng K Evans M Fisher J. A prospective study of predictors of prolonged hospital stay and disability after stroke. J Clin Neurosci. (2003) 10:665–9. 10.1016/j.jocn.2002.12.001 14592613

[5] Ji RJ Shen HP Pan YS Wang PL Liu GF Wang YL. novel risk score to predict pneumonia after acute ischemic stroke. Stroke. (2013) 44:1303. 10.1161/STROKEAHA.111.000598 23482598

[6] Hoffmann S Malzahn U Harms H Koennecke HC Berger K Kalic M. Development of a clinical score (A2DS2) to predict pneumonia in acute ischemic stroke. Stroke. (2012) 43:2617–23. 10.1161/STROKEAHA.112.653055 22798325

[7] Feng HX Cheng Y Zhu W Jiang LL Dong XF Gui Q. T-lymphocyte subsets as a predictive biomarker for stroke-associated pneumonia. Am J Transl Res. (2018) 10:4367–75. 30662678 PMC6325496

[8] Vogelgesang A Grunwald U Langner S Jack R Broker BM Kessler C. Analysis of lymphocyte subsets in patients with stroke and their influence on infection after stroke. Stroke. (2008) 39:237–41. 10.1161/STROKEAHA.107.493635 18048864

[9] Nam KW Kim TJ Lee JS Kwon HM Lee YS Ko SB. High neutrophil-to-lymphocyte ratio predicts stroke-associated pneumonia. Stroke. (2018) 49:1886–92. 10.1161/STROKEAHA.118.021228 29967014

[10] Wu G Gong S Cai H Ding Y. The availability, price and affordability of essential antibacterials in Hubei province, China. BMC health Serv Res. (2018) 18:1013. 10.1186/s12913-018-3835-x 30594189 PMC6310993

[11] Yezli S Li H. Antibiotic resistance amongst healthcare-associated pathogens in China. Inte J Antimicrob Agents. (2012) 40:389–97. 10.1016/j.ijantimicag.2012.07.009 22999767 PMC7135469

[12] Horan TC Gaynes RP Martone WJ Jarvis WR Emori TG. CDC definitions of nosocomial surgical site infections, 1992: a modification of CDC definitions of surgical wound infections. Infect Control Hosp Epidemiol. (1992) 13:606–8. 10.1017/S0195941700015241 1334988

[13] Smith CJ Kishore AK Vail A Chamorro A Garau J Hopkins SJ. Diagnosis of stroke-associated pneumonia: recommendations from the pneumonia in stroke consensus group. Stroke. (2015) 46:2335–40. 10.1161/STROKEAHA.115.009617 26111886

[14] Westendorp WF Vermeij JD Zock E Hooijenga IJ Kruyt ND Bosboom HJLW. The Preventive Antibiotics in Stroke Study (PASS): a pragmatic randomised open-label masked endpoint clinical trial. Lancet. (2015) 385:1519–26. 10.1016/S0140-6736(14)62456-9 25612858

[15] Suda S Aoki J Shimoyama T Suzuki K Sakamoto Y Katano T. Stroke-associated infection independently predicts 3-month poor functional outcome and mortality. J Neurol. (2018) 265:370–5. 10.1007/s00415-017-8714-6 29249057

[16] Minnerup J Wersching H Brokinkel B Dziewas R Heuschmann PU Nabavi DG. The impact of lesion location and lesion size on poststroke infection frequency. J Neurol Neurosur Ps. (2010) 81:198–202. 10.1136/jnnp.2009.182394 19726403

[17] Qin XH Li JP Spence JD Zhang Y Li YB Wang XB. Folic acid therapy reduces the first stroke risk associated with hypercholesterolemia among hypertensive patients. Stroke. (2016) 47:2805–12. 10.1161/STROKEAHA.116.014578 27729579

[18] Berglund L. Evaluation and treatment of hypertriglyceridemia: an endocrine society clinical practice guideline (vol 97, pg 2969. 2012). J Clin Endocr Metab. (2015) 100:4685. 10.1210/jc.2011-3213 22962670 PMC3431581

[19] Kwon HM Jeong SW Lee SH Yoon BW. The pneumonia score: a simple grading scale for prediction of pneumonia after acute stroke. Am J Infect Control. (2006) 34:64–8. 10.1016/j.ajic.2005.06.011 16490608

[20] Chumbler NR Williams LS Wells CK Lo AC Nadeau S Peixoto AJ. Derivation and validation of a clinical system for predicting pneumonia in acute stroke. Neuroepidemiology. (2010) 34:193–9. 10.1159/000289350 20197702 PMC2883837

[21] Walter U Knoblich R Steinhagen V Donat M Benecke R Kloth A. Predictors of pneumonia in acute stroke patients admitted to a neurological intensive care unit. J Neurol. (2007) 254:1323–9. 10.1007/s00415-007-0520-0 17361338

[22] Brogan E Langdon C Brookes K Budgeon C Blacker D. Respiratory infections in acute stroke: nasogastric tubes and immobility are stronger predictors than dysphagia. Dysphagia. (2014) 29:340–5. 10.1007/s00455-013-9514-5 24445382

[23] Warusevitane A Karunatilake D Sim J Lally F Roffe C. Safety and effect of metoclopramide to prevent pneumonia in patients with stroke fed via nasogastric tubes trial. Stroke. (2015) 46:454–60. 10.1161/STROKEAHA.114.006639 25516196

[24] Hinchey JA Shephard T Furie K Smith D Wang D Tonn S. Formal dysphagia screening protocols prevent pneumonia. Stroke. (2005) 36:1972–6. 10.1161/01.STR.0000177529.86868.8d 16109909

[25] Giede-Jeppe A Bobinger T Gerner ST Sembill JA Sprugel MI Beuscher VD. Neutrophil-to-Lymphocyte ratio is an independent predictor for in-hospital mortality in spontaneous intracerebral hemorrhage. Cerebrovasc Dis. (2017) 44:26–34. 10.1159/000468996 28419988

[26] Xue J Huang WS Chen XL Li Q Cai ZY Yu T. Neutrophil-to-lymphocyte ratio is a prognostic marker in acute ischemic stroke. J Stroke Cerebrovasc. (2017) 26:650–7. 10.1016/j.jstrokecerebrovasdis.2016.11.010 27955949

[27] Qun S Tang Y Sun J Liu Z Wu J Zhang J. Neutrophil-To-lymphocyte ratio predicts 3-month outcome of acute ischemic stroke. Neurotox Res. (2017) 31:444–52. 10.1007/s12640-017-9707-z 28181171

[28] Kim JY Park J Chang JY Kim SH Lee JE. Inflammation after ischemic stroke: the role of leukocytes and glial cells. Exp Neurobiol. (2016) 25:241–51. 10.5607/en.2016.25.5.241 27790058 PMC5081470

[29] de Jager CPC Wever PC Gemen EFA Kusters R van Gageldonk-Lafeber AB van der Poll T. The neutrophil-lymphocyte count ratio in patients with community-acquired pneumonia. PLoS ONE. (2012) 7:e46561. 10.1371/journal.pone.0046561 23049706 PMC3462173

[30] Zahorec R. Ratio of neutrophil to lymphocyte counts–rapid and simple parameter of systemic inflammation and stress in critically ill. Bratisl Lek Listy. (2001) 102:5–14. 11723675

[31] Joshi VD Kalvakolanu DV Cross AS. Simultaneous activation of apoptosis and inflammation in pathogenesis of septic shock: a hypothesis. FEBS Lett. (2003) 555:180–4. 10.1016/S0014-5793(03)01271-7 14644412

[32] Prass K Meisel C Hoflich C Braun J Halle E Wolf T. Stroke-induced immunodeficiency promotes spontaneous bacterial infections and is mediated by sympathetic activation reversal by poststroke T helper cell type 1-like immunostimulation. J Exp Med. (2003) 198:725–36. 10.1084/jem.20021098 12939340 PMC2194193

[33] Shim R Wong CHY. Ischemia, immunosuppression and infection-tackling the predicaments of post-stroke complications. Int J Mol Sci. (2016) 17:64. 10.3390/ijms17010064 26742037 PMC4730309

[34] de Jager CPC van Wijk PTL Mathoera RB de Jongh-Leuvenink J van der Poll T Wever PC. Lymphocytopenia and neutrophil-lymphocyte count ratio predict bacteremia better than conventional infection markers in an emergency care unit. Crit Care. (2010) 14:R192. 10.1186/cc9309 21034463 PMC3219299

[35] Nordestgaard BG. Triglyceride-rich lipoproteins and atherosclerotic cardiovascular disease: new insights from epidemiology, genetics, and biology. Circ Res. (2016) 118:547–63. 10.1161/CIRCRESAHA.115.306249 26892957

[36] Antonios N Angiolillo DJ Silliman S. Hypertriglyceridemia and ischemic stroke. Eur Neurol. (2008) 60:269–78. 10.1159/000157880 18824854

[37] Grundy SM Stone NJ Bailey AL Beam C Birtcher KK Blumenthal RS. (2018). AHA/ACC/AACVPR/AAPA/ABC/ACPM/ADA/AGS/APhA/ASPC/NLA/PCNA guideline on the management of blood cholesterol: a report of the American college of cardiology/American heart association task force on clinical practice guidelines. Circulation. (2019) 139:e1082–e143. 10.1161/CIR.0000000000000700 30586774 PMC7403606

[38] Aung T Halsey J Kromhout D Gerstein HC Marchioli R Tavazzi L. Associations of omega-3 fatty acid supplement use with cardiovascular disease risks: meta-analysis of 10 trials involving 77 917 individuals. JAMA Cardiol. (2018) 3:225–34. 10.1001/jamacardio.2017.5205 29387889 PMC5885893

[39] Chan MC Lin CH Kou YR. Hyperlipidemia in COPD is associated with decreased incidence of pneumonia and mortality: a nationwide health insurance data-based retrospective cohort study. Int J Chronic Obstr. (2016) 11:1053–9. 10.2147/COPD.S102708 27274227 PMC4876799

[40] Ouchi N Kihara S Funahashi T Matsuzawa Y Walsh K. Obesity, adiponectin and vascular inflammatory disease. Curr Opin Lipidol. (2003) 14:561–6. 10.1097/00041433-200312000-00003 14624132

[41] Katsiki N Mantzoros C Mikhailidis DP. Adiponectin, lipids and atherosclerosis. Curr Opin Lipidol. (2017) 28:347–54. 10.1097/MOL.0000000000000431 28463859

[42] van de Woestijne AP Monajemi H Kalkhoven E Visseren FLJ. Adipose tissue dysfunction and hypertriglyceridemia: mechanisms and management. Obes Rev. (2011) 12:829–40. 10.1111/j.1467-789X.2011.00900.x 21749607

[43] Bourlier V Zakaroff-Girard A Miranville A De Barros S Maumus M Sengenes C. Remodeling phenotype of human subcutaneous adipose tissue macrophages. Circulation. (2008) 117:806–15. 10.1161/CIRCULATIONAHA.107.724096 18227385

[44] Lau WB Ohashi K Wang YJ Ogawa H Murohara T Ma XL. Role of adipokines in cardiovascular disease. Circ J. (2017) 81:920–8. 10.1253/circj.CJ-17-0458 28603178

[45] Nakamura K Fuster JJ Walsh K. Adipokines: a link between obesity and cardiovascular disease. J Cardiol. (2014) 63:250–9. 10.1016/j.jjcc.2013.11.006 24355497 PMC3989503

[46] Walson JL Berkley JA. The impact of malnutrition on childhood infections. Curr Opin Infect Dis. (2018) 31:231–6. 10.1097/QCO.0000000000000448 29570495 PMC6037284

[47] Verhagen LM Gomez-Castellano K Snelders E Rivera-Olivero I Pocaterra L Melchers WJG. Respiratory infections in enepa amerindians are related to malnutrition and streptococcus pneumoniae carriage. J Infect. (2013) 67:273–81. 10.1016/j.jinf.2013.06.010 23796866 PMC7173337

[48] Li Y Zhang Y Ma L Niu X Chang J. Risk of stroke-associated pneumonia during hospitalization: predictive ability of combined A(2)DS(2) score and hyperglycemia. BMC Neurol. (2019) 19:298. 10.1186/s12883-019-1497-x 31766993 PMC6876087

[49] Balachandran VP Gonen M Smith JJ DeMatteo RP. Nomograms in oncology: more than meets the eye. Lancet Oncol. (2015) 16:E173–E80. 10.1016/S1470-2045(14)71116-7 25846097 PMC4465353

[50] Jin R Yang G Li G. Inflammatory mechanisms in ischemic stroke: role of inflammatory cells. J Leukoc Biol. (2010) 87:779–89. 10.1189/jlb.1109766 20130219 PMC2858674

[51] Kammersgaard LP Jorgensen HS Nakayama H Reith J Raaschou HO Olsen TS. Leukocytosis in acute stroke: relation to initial stroke severity, infarct size, and outcome: the copenhagen stroke study. J Stroke Cerebrovasc Dis. (1999) 8:259–63. 10.1016/S1052-3057(99)80076-7 17895174

